# Topical review: Lactation and use of DMTs in women with MS

**DOI:** 10.1177/13524585241257843

**Published:** 2024-09-30

**Authors:** Stephanie Hsu, Ayushi Balan, Riley Bove

**Affiliations:** University of California San Francisco, San Francisco, CA, USA; University of California San Francisco, San Francisco, CA, USA; University of California San Francisco, San Francisco, CA, USA

**Keywords:** Lactation, amenorrhea, mother–infant dyad, breastmilk, reproductive safety

## Abstract

One in three females with multiple sclerosis (MS) becomes pregnant after diagnosis. In the postpartum period, there is a risk of rebound inflammatory activity. This risk can likely be reduced with breastfeeding, as well as with early initiation of effective therapies that have low therapeutic lag. To guide patients in their choices surrounding breastfeeding and MS therapies, clinicians must be familiar with how best to protect against relapses, to ensure infant safety, and to support breastfeeding choices. This topical review provides a broad framework on lactation in women with MS. It seeks to reframe guidelines around caring for the maternal–infant dyad, and for diverse populations living with MS. It also provides updated data on the effects of lactation in women with MS and the limited data on transfer of disease-modifying therapies (DMTs) into breastmilk. The ultimate goal is to support informed shared decision-making between clinicians and patients regarding breastfeeding during the high-risk postpartum period.

## Introduction

Two-thirds of all individuals diagnosed with multiple sclerosis (MS) are females of reproductive age.^1,2^ After MS diagnosis, approximately one in three female patients become pregnant.^3^ For these patients, breastfeeding represents an important counseling topic. A number of factors influence shared decision-making around breastfeeding, including patient personal preference and logistical support, relevance of breastfeeding to the childbearing and maternal experience, education about the benefits of breastfeeding and access to lactation support, maternal and infant physical/medical factors, and planning surrounding MS disease-modifying therapy (DMT) resumption.

Historically, people with MS have been cautiously counseled against DMT use during breastfeeding. Key stakeholders—pharmaceutical companies and neurologists—have not, with some notable exceptions,^4,5^ focused enough on specific DMT transfer during lactation. Instead, the discussion has historically been centered around avoiding potential infant harm. Consequentially, women with MS have often been advised to either forego breastfeeding entirely, or to resume DMTs only at weaning in order to limit inadvertent infant exposure. While avoiding potential infant harm is of paramount importance, this singular focus has sidestepped the prevention of maternal MS disease activity and the potential benefits of breastfeeding.

Clinicians are now beginning to reframe clinical decision-making and risk-benefit perspectives on the totality of the mother–infant dyad, explicitly considering the physical and mental health of the birthing person. In addition, data have begun to emerge about extent of DMT transfer into breastmilk and the outcomes of potentially exposed infants, allowing both clinicians and patients to participate in more informed, shared decision-making.

The goal of this topical review is to provide an overview for practicing neurologists on lactation; MS management; and maternal, newborn, and dyadic well-being. To accomplish this, a topical review of the literature concerning lactation in women with MS was conducted using PubMed. No restrictions were placed on country or article type. The search included terms such as, but not limited to, the following: [lactation] or [breastfeeding] AND [multiple sclerosis] or [chronic disease] OR [disease modifying therapies including each product named in Table 1], [postpartum] OR [pregnancy] AND [multiple sclerosis]. To augment ongoing periodic reviews on lactation safety of MS medications, a major focus was on providing a comprehensive perspective on lactation.

**Table 1.** Summary of breastmilk transfer, infant safety, and regulatory data available for DMTs during lactation (15 July 2023).

	Breastfeeding transfer data: concentrations and/or RID measured in mature breastmilk	Clinical data relating to breastmilk safety in infants	Label recommendations for lactation
First-line self-injectables: large molecules with low oral bioavailability can be used during lactation			
Glatiramer acetate	RID < 1% (*N* = 17)^58^	At 18 months, typical development (*N* = 60)^59^	FDA: Consider maternal clinical needs and potential adverse effects^60^ EMA: Can be used^61^
Interferon beta	RID < 1% (*N* = 11)^4,62^	At 12 months, typical development (*N* = 39)^63^	FDA: Consider maternal clinical needs and potential adverse effects^64^ EMA: Can be used^65^
Oral medications: low molecular weight, orally bioavailable molecules should be avoided during lactation until further data are available			
Cladribine	RID 3%–4.8% (*N* = 3)^66^	—	FDA: Contraindicated during breastfeeding and for at least 1 week after treatment discontinuation^67^ EMA: Contraindicated during breastfeeding and for at least 1 week after treatment discontinuation^68^
Dimethyl fumarate	RID < 1% (*N* = 2)^69^	—	FDA: Consider maternal clinical needs and potential adverse effects^70^ EMA: Should be discontinued^71^
Teriflunomide	No human data reported Animals: does transfer into milk	—	FDA: Should be discontinued^72^ EMA: Should be discontinued^73^
Fingolimod	No human data reported Animals: does transfer into milk	—	FDA: Consider maternal clinical needs and potential adverse effects^74^ EMA: Should be discontinued^75^
Ozanimod	No human data reported Animals: does transfer into milk	—	FDA: Consider maternal clinical needs and potential adverse effects^76^ EMA: Should be discontinued^77^
Siponimod	No human data reported Animals: does transfer into milk	—	FDA: Consider maternal clinical needs and potential adverse effects^78^ EMA: Should be discontinued^79^
Ponesimod	No human data reported Animals: does transfer into milk	—	FDA: Consider maternal clinical needs and potential adverse effects^80^ EMA: Should be discontinued^81^
Monoclonal antibodies: molecules with low physiologic IgG transfer into breastmilk and low oral bioavailability can be continued during lactation			
Alemtuzumab	No human data reported Animals: does transfer into milk	Caution advised due to potential transfer of thyroid antibodies	FDA: Consider maternal clinical needs and potential adverse effects^82^ EMA: Lactation contraindicated until 4 months after last treatment; should also consider potential immunological benefits of breastmilk to infant^83^
Natalizumab	RID < 1% (*N* = 15)^84–86^ RID up to 5.3% (*N* = 1)^87^ Maximal concentration of 0.41 µg/m (*N* = 4)^5^	At 12 months, typical development (*N* = 17)^86^ Not detectable in infant serum 2 days after maternal infusion (*N* = 2)^86,88^	FDA: Consider maternal clinical needs and potential adverse effects^89^ EMA: Should be discontinued^90^
Rituximab	RID < 1% (*N* = 21;^49^ *N* = 6,^91^ *N* = 1,^92^ and *N* = 1^93^)	At 12 months, typical development (*N* = 33)^49,86,92^ Normal B cells reported in 11/11^49,86,91^ Undetectable concentration/levels in seven lactating infants^91,92^	FDA: Lactation contraindicated until 6 months after last treatment^94^ EMA: Lactation contraindicated until 6 months after last treatment^95^
Ocrelizumab	RID < 1% in serial samples collected from 30 women^49^	After 12 months, no negative outcomes observed in 32 infants breastfed after maternal treatment versus infants weaned^49^ Normal B cells reported in 5/5^49,86^ Ongoing SOPRANINO study is prospectively evaluating safety outcomes in infants breastfed by mothers on ocrelizumab^96^	FDA: Consider maternal clinical needs and potential adverse effects^97^ EMA: Should be discontinued during breastfeeding^98^
Ofatumumab	No human data reported Animals: does transfer into milk	N/A	FDA: Consider maternal clinical needs and potential adverse effects^99^ EMA: Avoid early postpartum; mature milk could be used during breastfeeding, if clinically needed^100^
Ublituximab	No human data reported Animals: does transfer into milk	N/A	FDA: Consider maternal clinical needs alongside potential adverse effects^101^ EMA: Avoid early postpartum; could be used during breastfeeding, if clinically needed^102^

DMT: disease-modifying therapy; RID: relative infant dose; FDA: Federal Drug Administration; EMA: European Medicines Agency.

## Lactation: overview of common terminology and practices

### Terminology

Exclusive breastfeeding refers to breastfeeding when the infant only receives breastmilk, without any additional fluids or foods other than medications, vitamins, and minerals.^6^ Semi-exclusive, or non-exclusive breastfeeding, includes the addition of foods and fluids to the infant’s diet along with breastmilk. Both the World Health Organization (WHO) and the American Academy of Pediatrics (AAP) recommend exclusive breastfeeding for the first 6 months of life.

### Heterogeneity in practices and choices

Around the world, approximately 68% of women breastfeed for at least 1 year, with that percentage dropping to 44% by 2 years. But only 44% of infants are exclusively breastfed until 6 months of age.^7^ It is estimated that approximately 83% of American women breastfeed their infants, with that rate decreasing to 58% around 6 months.^8^ Across other Western countries, breastfeeding rates vary greatly, from 46% to 95%. The Center for Disease Control and Prevention (CDC) recommends breastfeeding, irrespective of disease status.^9^ Chronic conditions may increase the risk of poor pregnancy outcomes—including preterm birth, cesarean section, and other adverse perinatal outcomes.^10^

This heterogeneity can be attributed to societal (cultural and religious norms governing maternal preferences and maternal identity,^11^ parental leave policies, lactation support available and affordable), individual (personal goals and preference, income, employment, education, practical support available), and medical (history of breast surgery, infant conditions influencing latching, use of medications unsafe during lactation) factors, among others.^12^

### Potential challenges in the MS population

Women with chronic conditions may experience greater rates of breastfeeding challenges, such as ineffective latch or delayed and/or low lactation levels.^13^ Delayed milk production, in turn, can lead to earlier exclusive breastfeeding cessation.^14^ The presence, or extent, of an impact of MS on delayed lactogenesis is not well characterized. Difficulty with breastfeeding has been identified as a risk factor for peripartum depression in mothers with MS,^15^ underscoring the interrelated nature of lactation, MS, and peripartum mental health.^15^ Along with direct lactation challenges, breastfeeding disrupts maternal sleep, potentially contributing to fatigue and other effects on mothers’ quality of life;^16,17^ indeed, fatigue represents a major domain of “invisible” MS-related disability. Breastfeeding is also time consuming, which can add to the burden that people with MS experience in managing their daily lives. Maternal MS disease severity may also pose challenges, with higher disease activity pre-pregnancy associated with lower breastfeeding rates^18,19^—underscoring the perceived tension between continuing breastfeeding and the need for disease management. Given these challenges, breastfeeding may not represent the ideal choice for all women with MS. In cases where individuals do not intend to breastfeed, supportive statements such as “fed is best” as well as attention to specific contributing factors (fatigue, return to work) can be utilized to support their decision-making.

## Lactation: overview of physiology

### Physiology

Lactation occurs in several stages, which differ in immunological and nutritional properties. Human colostrum, produced during breastfeeding in the first five days postpartum, is high in immunologic components, such as secretory IgA, leukocytes, lactoferrin, and growth factors, and has a relatively low concentration of lactose.^20^ Milk is “transitional” until about 15 days, when it is considered “mature” as the tight junctions in the mammary epithelium close, yielding more nutritionally dense milk with a higher lactose concentration.^20,21^ Human milk is fully mature at 6 weeks postpartum. Bioactive components in breastmilk can be produced and secreted by the mammary epithelium, or can travel from maternal serum through the mammary epithelium via receptor-mediated transport.^20,21^

### Maternal hormonal regulation

Hormones associated with lactation have notable physiological effects on mothers. Progesterone levels decrease following delivery, triggering lactation and potentially resulting in antidepressant and anxiolytic effects over time.^22,23^ Levels of the pregnancy estrogen estriol, as well as other estrogens, also decrease. After the drop in progesterone levels, prolactin levels increase, in turn promoting the development of alveoli in the mammary gland. Prolactin acts on the nervous system to induce an increase in nursing behavior, food intake, and oxytocin release during lactation,^22^ that is, the “bonding hormone.” The high levels of both prolactin and oxytocin during lactation have been reported to play a protective role against peripartum mood disorders (depression, anxiety, and irritability) and may also improve stress reactivity for mothers.^24^ Peptide hormones and glucocorticoids secreted during lactation, such as insulin, IGF1, leptin, and adiponectin, also show important associations with maternal metabolism, mammary calcium-sensing receptor activity, obesity, and stress.^23^ Exclusive breastfeeding, distinct from semi-exclusive breastfeeding, maintains high prolactin levels and low luteinizing hormone levels.^25^ It also maintains lactational amenorrhea.^25^ Altogether, as is also suggested by studies of immune responsiveness to other hormonal changes (e.g. menses^26^), and still incompletely described in MS,^27^ these widespread hormonal changes in the postpartum period likely have downstream effects on cytokines regulating immune activity.

## Lactation: overview of benefits for mother, infant, and the dyad

### Maternal impacts

#### General maternal benefits

The benefits of breastfeeding include reduced risks of ovarian and breast cancer, and of cardiometabolic outcomes (obesity, hypertension, hyperlipidemia, type 2 diabetes, myocardial infarction, cardiovascular disease).^28–31^ During pregnancy, insulin production and resistance increase, as do visceral fat and lipid levels in the blood.^32^ Without lactation, these effects persist longer postpartum, and maternal metabolism is slower to revert to baseline.^33^

However, baseline maternal metabolism, as well as conditions such as perinatal depression and anxiety disorders, may affect lactation success.^34^

Breastfeeding also appears to be associated with lower risk of peripartum depression.^35–38^ Distinguishing its effects on mood in women with MS is more complicated. Individuals with MS have greater baseline prevalence of depression and anxiety^34,39^ and specific studies report greater prevalence of peripartum and postpartum depression (PPD) in women with MS compared to those without MS.^34,39^ Conversely, as noted above, breastfeeding difficulties may contribute to a greater risk of PPD in women with MS.^15^ Therefore, it is not clear whether lower depression in women with MS may reflect the benefits of breastfeeding or the absence of breastfeeding difficulties. For women with MS who do plan to breastfeed, adequate lactation support may be essential to promoting their goals and reducing distress and possibly depression.

#### Protective effects against postpartum inflammatory activity

The early postpartum period is marked by an elevated risk of MS inflammatory activity (clinical relapses and/or new magnetic resonance imaging (MRI) activity) after relative quiescence during the immunotolerant state of pregnancy, typically during the last trimester.^40^ Since the incidence, pace and duration of lactation varies substantially in different settings, it is therefore not surprising that there has been heterogeneity in studies identifying a protective effect of breastfeeding as well as differentiating between the potential effects of exclusive and non-exclusive breastfeeding. Some possible confounding arises from the fact that women with elevated disease activity pre-conception—itself a risk factor for postpartum inflammatory activity,^41,42^ may be advised to forego breastfeeding in favor of early DMT initiation. This would lead to over-representation of women with low disease activity in the breastfeeding groups and an apparent protective effect.

Nonetheless, on meta-analyses, breastfeeding does appear associated with lower risk of relapses in the early postpartum period, with a stronger association seen with exclusive breastfeeding.^40,43^ To quantify this effect, a recent meta-analysis from 2023 reported that exclusive breastfeeding was associated with a lower risk ratio for relapses (0.39, 95% confidence interval (CI) = 0.18 to 0.86; *p* = 0.02) in the first 6 months postpartum compared with not breastfeeding. Those who breastfed exclusively also had a lower risk ratio for relapses (0.55, 95% CI = 0.31 to 0.97; *p* *=* 0.04), compared with those who breastfed non-exclusively.^40^ This supports a causative mechanism since breastfeeding status (yes/no), but not exclusivity, would reflect clinical decision-making. Furthermore, exclusive breastfeeding also appears associated with a reduced risk of new MRI lesions postpartum.^42^ The extent to which reducing inflammatory activity improves quality of life in the postpartum period has not been quantified.

### Newborn health

Breastfeeding is associated with reduced infections in infants, including otitis media, gastroenteritis, and pneumonia. It is also associated with reduced rates of sudden infant death syndrome (SIDS), and of childhood obesity, diabetes, leukemia, asthma (reviewed in Stuebe and Rich-Edwards^33^). Interesting data also link breastfeeding with reduced rates of autoimmune disorders in adulthood including diabetes mellitus type 1, MS, celiac disease, and systemic lupus erythematosus (reviewed in previous studies^44–47^).

### Maternal–infant dyad

For the maternal–infant dyad, breastfeeding represents a culturally sanctioned and physiologically facilitated type of bonding. A greater extent of breastfeeding difficulties (breast engorgement, non-latching) during breastfeeding has been linked with more difficult bonding.^48^

## DMTs during lactation

### General principles

Despite the many health benefits of breastfeeding, its timing coincides during a period of susceptibility to increased inflammatory activity. As such, it follows that in women with MS who choose to breastfeed, one of the important questions for postpartum MS management is whether, and when, to resume DMT. As the number of DMTs continues to increase, so too does the need for greater knowledge in this arena. Recent studies suggest that early initiation of specific high-efficacy therapies in postpartum may play a protective role against relapses,^42,49^ although the data remain scant. For instance, in a study of 57 women who received anti-CD20 monoclonal antibodies in the first 12 months postpartum, only two women experienced relapses after postpartum infusion; and in the subgroup of 14 women treated with antiCD20 within 6 months of conception and within 5 weeks of delivery, 0 experienced relapses over the first year postpartum.^50^ Another study of natalizumab initiated early postpartum did not identify a protective effect against early postpartum relapses.^51^ The utilization of scheduled methylprednisolone as an alternative to DMTs as prophylaxis against relapses during this period of heightened inflammatory activity postpartum is not favored, as it showed no efficacy (1 g per month intravenously from delivery up to 6 months).^52^

When evaluating the safety of a DMT during lactation, factors to consider for each DMT are (1) biological properties that would influence breastmilk transfer, (2) existing evidence relating to transfer to breastmilk, and (3) potential impact to infant if exposed to the drug. Regarding factor (1), that is, likelihood of transfer, one of the most critical factors is the molecular weight of the drug: drugs with larger molecular weight are less likely to be able to transfer into breastmilk.^53^ Almost all drugs are transferred between maternal plasma and breastmilk via passive diffusion, and as such, concentration of drug in maternal plasma is also an important element in potential drug transfer.^54^ Other relevant factors, such as dosage timing, maternal protein plasma binding, and drug lipid solubility, are reviewed by Hotham and Hotham^54^ and Rowe et al.^53^ Infant exposure to a DMT can be evaluated by calculating the relative infant dose (RID), which is the weight-and-time-adjusted percentage of maternal dose. Typically, an RID under 10% is considered compatible with lactation,^55^ although the oral bioavailability of the drug in the infant’s gastrointestinal tract,^53^ the potential toxicity of the drug, and the infant’s age (newborns are less able to able to metabolize drugs),^56,57^ are also key to understanding potential drug absorption, metabolism, and impact.

### DMTs in lactation: current overview

A review of the data on breastmilk transfer, infant outcomes, and regulatory guidance regarding each DMT and lactation is provided in Table 1. There is substantial heterogeneity in the information available for each product, but transfer of most medications into breastmilk appears overall low. These can be summarized as follows, for the case of maternal treatment once milk is mature (i.e. 2 weeks).

- The first-line injectables such as interferon beta and glatiramer acetate are considered safe for use during pregnancy and lactation, given low RID, low oral bioavailability, and reassuring infant outcomes.^4,58,59,103^
- Overall, oral agents should be avoided when breastfeeding—given their smaller size and oral bioavailability if ingested. While early data suggest that the RID for both cladribine and dimethyl fumarate is < 10%, the data are extremely limited to date, and longer-term safety outcomes are needed.^69^
- Monoclonal antibodies (IgG) have high molecular weight, do not transfer into breastmilk at significant concentrations physiologically, and have low oral bioavailability. RID for all IgG1 monoclonals is reported to be < 1%^104^ and this is corroborated for those used to treat MS (see Table 1). While data exist for rituximab, ocrelizumab, and natalizumab, they are lacking for ofatumumbab, ublituximab, and alemtuzumab. Residual questions include whether thyroid antibodies can be transferred in women on alemtuzumab, and whether B-cell depletion might influence the immunological effects of breastfeeding (e.g. IgA levels) independently of the question of breastmilk transfer.
- Should patients experience a clinical relapse, transfer of methylprednisolone is also minimal (< 1%, *N* = 12)^105^ and can be used. No adverse effects were reported in breastfed infants over a 6–24-month follow-up period.^106^
- Transfer of pre-medications (methylprednisolone and antihistamines) used for some infusions is also minimal, but a short (2–4 hour) period of pumping and discarding can be considered to further reduce risk of transfer.^105,106^

### Reconciling heterogeneous sources regarding safety of medications during lactation

To update clinicians and ultimately optimize shared decision-making, comprehensive reviews,^107–111^ sometimes multidisciplinary,^112–114^ are published periodically, providing updated data and guidance regarding DMT use during lactation. For clinicians and patients alike, obtaining updated and consistent information can be challenging. Many patients receive inconsistent guidance from neurologists, pediatricians, and other professionals. To take the example of IgG monoclonal antibodies, there is no formal position from neurological societies. However, the American College of Rheumatology, American Gastroenterological Association, and AAP all opine that it is “safe” to initiate or continue treatment while breastfeeding.^115–117^ Alternatively, relying on regulatory product labels alone (FDA, Federal Drug Administration and EMA, European Medicines Agency) can result in physiologically inconsistent practices that lag behind clinical data. A recently published study highlighted that the FDA and EMA differ in their pregnancy labeling by approximately 68% and lactation labeling by around 71%, and that only 16% of lactation labels included human data.^118^ For MS DMTs, this heterogeneity is clearest in the guidance for use of B cell-depleting anti-CD20 monoclonal antibodies (see Table 1).

To keep pace with accumulating data, databases such as the National Library of Medicine and National Institutes of Health LactMed database (United States) can bridge the gap. LactMed is a publicly and freely available online database for both clinicians and patients, compiling comprehensive literature-based safety data on medications most commonly used by lactating individuals. It is updated regularly and can be downloaded via mobile app. In Europe, e-lactancia is a Spanish website that compiles concise and patient-facing recommendations regarding breastfeeding from the EMA, LactMed, and more. The website also classifies each medication into a lactation risk category. Other resources are summarized elsewhere (see Table 2).^119^ These sites are not without limitations in the quality or relevance of research data available: only 2% medications have comprehensive research study data on maternal drug levels, infant drug levels, effects on infants, and effects on lactation.^120^ Therefore, clinicians must familiarize themselves with general principles, and in areas of data paucity, align with a patient’s other clinicians (pediatric, obstetric) to harmonize messaging about product safety and recommendations.

**Table 2.** Use of symptomatic medications during lactation.

Name of medication	Mood	Pain	Fatigue	Spasticity	Ambulation	Urinary infections	Other
SSRIs (sertraline, paroxetine)	Continue						
TCAs (amitriptyline/nortriptyline)	Continue	Continue					
Duloxetine	Continue	Continue					
Clonazepam and diazepam	Anxiety: avoid						
Carbamazepine		Continue					Seizures: continue
Ibuprofen		Continue					
Paracetamol		Continue					
Gabapentin		Continue					
Tramadol		Continue with caution					
Pregabalin		Avoid					
Sativex		Avoid					
Amantadine			Avoid				
Modafinil			Avoid				
Stimulants			Continue with caution				
Baclofen				Caution			
Tizanidine				Avoid			
Fampridine					Avoid		
Amoxicillin/clavulanic acid						Continue	
Trimethoprim						Continue	
Nitrofurantoin						Continue; avoid only if infant has G6PD deficiency or jaundice	

Source: Modified from the study by Iyer et al.^119^

SSRI: selective serotonin reuptake inhibitor; TCA: tricyclic antidepressant; G6PD: glucose-6-phosphate dehydrogenase.

## Lactation: evidence-based strategies to overcome heterogeneity in adoption

According to the WHO, if circumstances permit, breastfeeding for optimal health should be encouraged for at least 12 months, with the first 6 months being exclusive breastfeeding.^28^ As described above, however, breastfeeding may not represent the right choice for everyone. When patients do choose to breastfeed, lactation support can come through policy, dissemination of guidelines and materials, and formal lactation support and educational programming.

### Lactation consultants

While physicians, nurses, and other allied health professionals can support breastfeeding, lactation consultants and counselors are specifically trained to assist with different lactation problems and goals, and provide educational, logistical, and emotional support during pregnancy and lactation. Lactation consultants have been shown to increase rates of breastfeeding initiation and maintenance, including exclusive breastfeeding.^121,122^

### Disparities in lactation

Lactation support also should be targeted toward communities with lower breastfeeding rates, durations, and exclusivity. Indeed, a number of socioeconomic factors influence the incidence and duration of breastfeeding:

- Maternal factors, such as smoking, lower education attainment, having an unplanned pregnancy, higher body mass index (BMI), and anxiety;
- Obstetrical care-related factors, such as cesarean delivery and access to lactation education;^123,124^
- Socioemotional factors, such as maternal perception of having someone to turn to support the mother–infant dyad, or living in a safe neighborhood;
- Societal factors, such as “first food deserts” (areas with social, economic, cultural, and geographic contributors to lower rates of breastfeeding^125,126^) and lack of societal norms around breastfeeding.^122^ Varied acceptance and support of breastfeeding in workplaces also acts as a driver of disparity.^127^ Race/ethnicity disparities in the United States have specifically been noted. There is wide variability in cultural norms, expectations, and support for breastfeeding within “Black” and “Hispanic” populations in the United States,^128^ with both groups reported to have overall lower rates of breastfeeding. Multi-pronged strategies to mitigate these disparities include intentional clinical counseling about lactation with mothers, engagement of other supportive family members and friends when appropriate,^128^ explicit recommendations regarding support (peer-to-peer counseling, intergenerational emotional support^129^) and workplace strategies, and enhancing diversity among lactation support counselors, consultants, educators, and researchers.^128,129^

### Gender inclusivity

Maternal and gender identity are constructs distinct from the biological aspects of pregnancy and lactation. Expanding beyond studies largely performed in *cis*-women is important to inform on aspects relating to all breastfeeding individuals, as reviewed by Garcia-Acosta et al.^130^ Some clinical practices when caring for gender-diverse individuals may include considering impact of prior chest surgery, use of gender-inclusive terms such as chestfeeding, and assessing for social support and lactation support needs.

## Summary and future directions

Pregnancy and lactation represent distinct episodes in the lives of individuals with MS where distinct factors weigh into treatment planning, including maximizing the well-being of the mother–infant (or parent-infant) dyad. The postpartum period represents one of the highest risk periods for inflammatory activity, with new inflammatory activity on MRI noted in over half of all pregnancies historically. Yet patients have not typically received the highest standard or research or care to address their clinical needs or overarching life plans. Breastfeeding represents a goal for many patients with MS, serving important health benefits for mother and infant, both immediate (e.g. nutrition, immune protection), short-term (e.g. bonding and mental health), as well as over the lifespan (e.g. risk of metabolic and immune disarray). For women with MS, depending on the risk of relapse postpartum, it is possible to leverage principles of breastmilk physiology, product oral bioavailability, and real-world experience, to select from several treatment options. The first-line self-injectable therapies and monoclonal antibodies (antiCD20 or natalizumab) are likely safe, with caution still warranted for oral medications. Finally, lactation should be considered within the broader framework of postpartum care in women with MS, focusing on reducing maternal disease activity, promoting recovery through attention to mind (depression, anxiety, sleep) and physical (balance, bladder) rehabilitative needs, and supporting equity in access to evidence-based medicines.
